# Information Sharing in Neurosurgery Topics Among Pediatric Patients and Loved Ones Within the Reddit Community

**DOI:** 10.7759/cureus.56571

**Published:** 2024-03-20

**Authors:** Jocelyn To, Victoria J Horak, Donia Momen, Lekha Chirala, John Paul G Kolcun, Sandi Lam, Jeffrey Raskin

**Affiliations:** 1 Medicine, Chicago Medical School, Rosalind Franklin University of Medicine and Science, North Chicago, USA; 2 Neurological Surgery, Rush University Medical Center, Chicago, USA; 3 Pediatric Neurological Surgery, Ann and Robert H. Lurie Children's Hospital of Chicago, Chicago, USA; 4 Neurological Surgery, Northwestern University Feinberg School of Medicine, Chicago, USA

**Keywords:** family life, pediatric neurosurgery, patient perception, social media, reddit

## Abstract

Introduction

With crowd-sourced knowledge, patients arrive at their healthcare visits ready to play an active role. This exploratory study seeks to understand common concerns among patients and loved ones on Reddit, an anonymous internet forum. Ultimately, recognizing common concerns can aid providers in directing their conversations with patients.

Methods

Reddit posts in the "hot" tab of each subreddit were retroactively screened from September 1, 2022. Posts written within a five-year period were included. Posts by pediatric patients, loved ones, and pregnant patients experiencing the condition or whose fetus was diagnosed were included. Posts omitting the poster's age or individuals, outside of loved ones, who self-identified as over the age of 17 were excluded.

Results

A total of 12 subreddits and 286 posts were identified, with 37% of posts written by patients and 63% of posts written by a loved one. R/scoliosis patients (n=29) and r/epilepsy loved ones (n=28) sought the most health advice. The subreddit r/hydrocephalus comprised the most post-operative treatment symptom questions. The r/cerebralpalsy subreddit sought the most advice related to daily activities.

Discussion

Patients within r/scoliosis, r/hydrocephalus, and r/epilepsy are asking health-related questions. Hydrocephalus patients utilize anonymous internet responses to prepare for upcoming healthcare visits. Individuals in r/cerebralpalsy are utilizing Reddit as a method of communication; understanding how these patients interact with social media can guide software development tailored for online interactions. The anonymity of Reddit prevents us from understanding the diversity of posters.

Conclusion

Reddit is an avenue to disseminate correct information to pediatric patients and loved ones. Healthcare providers can use the information gathered to tailor their discussions better.

## Introduction

The internet and social media websites have shaped the way patients and their loved ones are learning about their illnesses. Social media platforms facilitate opportunities for anonymous information seeking regarding medical conditions. Today's patients and loved ones of all ages present for healthcare visits with crowd-sourced knowledge from the general populace [1-5]. These online sources promote autonomy for patients and their loved ones, and may supply more confidence; paradoxically, however, these sources lack peer review and could lead to the promulgation of misinformation, thus frustrating communication during a clinical appointment [1,6].

Patient and loved one information seeking via social media platforms highlights an opportunity for providers to address a gap in patient education. Elkarim et al. performed a descriptive study of Facebook, Twitter, and YouTube to understand social media usage for hydrocephalus [7]. They found that procedural treatments and surgical products were commonly searched topics and that users tended towards private groups over anonymous information-sharing opportunities.

Reddit is a popular social media platform used by 14% of teenagers aged 13-17 and overall by 18% of Americans in the United States [8,9]. Anonymous posting is a major feature underlying the use of Reddit and may provide a more comfortable environment for information seeking than during an office visit. A second unique feature of Reddit is the use of "subreddits" or subgroups of the Reddit community in which Reddit users, or "Redditors," can post questions, start discussions, or simply share information.

We perform a descriptive study of subreddits related to pediatric neurosurgical patients and their loved ones to understand if there is a clinician-directed educational opportunity using this platform.

## Materials and methods

With over 8.3 billion posts created in 2022 alone, Reddit was chosen as the sole social media site of interest due to the deficiency of publications studying the role of Reddit in the neurosurgical community and its growing popularity [8-10]. Once the platform was determined, subreddits were identified via specific search terms related to neurosurgical conditions (Appendix A). Subreddits were included in the study if a community existed for the condition searched (i.e., the search term “hydrocephalus” yielded the subreddit “r/hydrocephalus”). Reddit posts within the "hot" tab of each subreddit were screened starting from September 1, 2022. With the goal of 50 posts, posts were included in the study if they fell within the five-year study period. Posts reach the “hot” tab by being “upvoted,” or liked, and the hot tab is a collection of the most popular postings among the Reddit community at the time of search. It is often the page Redditors browse while utilizing the website.

The number of members per subreddit was collected on October 9, 2022. For subreddits with larger communities, such as r/epilepsy, a third-party website was used to find older posts that were within the time frame but exceeded Reddit’s 1000-post limit [11]. The Redditor's relationship to the patient was categorized as self (age <18 years), loved one, or pregnant person. Subreddits related to pediatric patients aged <18 were included; adult Redditors and posts related to adults with the conditions or subreddits without an indicated age <18 including unspecified "teenager" were excluded.

Posts were subcategorized within each subreddit and adapted from similar studies analyzing Reddit posts within subreddits [12-14]. After reviewing the content among posts, categories were created by JT. Characterization of the posts into categories was completed by four authors (JT, JH, LC, and DM). The posts were characterized into one category each. If there was conflict among categorization, the post was reviewed by a third party and a discussion was made among the three authors involved in that specific post categorization. The ages specified by posters were subdivided into groups by their developmental state: fetus, baby (0-1 year), toddler (13 months-2 years), early childhood (3-5 years), middle childhood (6-12 years), teenager (13-17 years), and pregnant mom with the condition. Posts were further divided into asking for social advice, asking for health advice, providing health education or equipment suggestions, or describing their personal experience. Social and health advice were further stratified into different categories.

This study was exempted from the Institutional Review Board due to the use of publicly available data.

## Results

Table 1 describes a total of 12 subreddits and 286 posts identified related to epilepsy (n=30,451), scoliosis (n=17,625), cerebral palsy (n=4,746), seizures (n=3,710), brain tumors (n=1,447), hydrocephalus (n=1,302), spina bifida (n=997), degenerative disc disease (n=666), Chiari malformation (n=426), brain aneurysm (n=358), ataxia (n=172), and herniated disc (n=38). The total number of members ranged from 38 to 30,451.

**Table 1:** Overall statistics and age breakdown of members collected on October 9, 2022

			Patients asking		Loved one asking					
Subreddit	Member count	Number of posts	Pregnant mom with condition	Teenager (13-17 y/o)	Fetus	Baby (0-1 y/o)	Toddler (13 mo-2 y/o)	Early childhood (3-5 y/o)	Middle childhood (6-12 y/o)	Teenager (13-17 y/o)
r/epilepsy	30,451	50	5	7	2	4	2	6	18	6
r/scoliosis	17,625	50	0	39	0	0	0	0	4	7
r/cerebralpalsy	4,746	50	0	10	0	6	9	17	6	2
r/seizures	3,710	24	0	6	0	0	4	3	7	4
r/braintumor	1,447	14	0	6	0	2	0	1	4	1
r/hydrocephalus	1,302	50	0	17	2	17	7	1	4	2
r/spina bifida	997	39	0	9	6	7	2	6	7	2
r/degendiscdisease	666	2	1	1	0	0	0	0	0	0
r/chiarimalformation	426	3	0	1	0	0	0	0	2	0
r/brainaneurysm	358	2	0	2	0	0	0	0	0	0
r/ataxia	172	1	0	0	0	0	0	0	0	1
r/herniateddisc	38	1	0	1	0	0	0	0	0	0
TOTAL, N (%)	61,938	286	6 (2.1%)	99 (34.6%)	10 (3.5%)	36 (12.6%)	24 (8.4%)	34 (11.9%)	52 (18.2%)	25 (8.7%)

Of the 286 posts, 105 posts were written by the patients themselves, and the remaining 181 posts were written by a loved one. Overall, the age group with the most common inquiries was teenagers (13-17 y/o), with 99 teenagers seeking information for themselves, and 25 loved ones seeking information related to a teenager. The demographics of posts made by loved ones were widely distributed, with the most common age group being middle childhood (18.2%).

Table 2 shows the majority of Redditors sought out health advice (63.3%, n=181), followed by social advice (23.8%, n=68). All subreddits primarily sought information regarding health advice (Table 3) except for r/cerebralpalsy, which focused on social advice (Appendix B, Table 10). Table 4 identifies within health-advice seeking, Redditors specifically inquired about treatment-related advice (n=63, 34.8%). Table 5 identifies a difference between patients and loved ones; most patients sought treatment-related advice (n=29, 46.0%), while loved ones asked 83.3% (n=35) of all questions related to disease progression. Treatment-related advice for loved ones was a close second among health-related categories (n=34, 54.0%). Overall, Tables 6, 7 show among treatment-related questions, patients and loved ones commonly had post-operative surgical questions (n=23, 36.5%). Table 8 shows among social advice for all information seekers, daily activities (n=31, 45.6%) was the most popular category. Table 9 identifies that loved ones asked 64.5% of all questions related to daily activities. Patients themselves, however, equally inquired about daily activities (n=11) and anxiety/depression (n=11).

**Table 2:** Overall breakdown of posts

Category	N (%)
Asking for health advice	181 (63.3%)
Asking for social advice	68 (23.8%)
Providing health education/suggesting equipment	5 (1.7%)
Personal experience/progress post	28 (9.8%)
Other	4 (1.4%)
Overall total	286

**Table 3:** Post categorizations broken down by author type: patient or loved one

	Patient, N (%)	Loved one, N (%)	Total, N
Asking for health advice	67 (37.0%)	114 (63.0%)	181
Asking for social advice	24 (35.3%)	44 (64.7%)	68
Providing health education/suggesting equipment	1 (20.0%)	4 (80.0%)	5
Personal experience/progress post	12 (42.9%)	16 (57.1%)	28
Other	1 (25.0%)	3 (75.0%)	4

**Table 4:** Subcategorization of the 181 posts seeking health-related information

Category	Total, N (%)
Is this symptom normal?	25 (13.8%)
Where do I get help? How do I speak to my doctor?	5 (2.8%)
What do these test results mean?	8 (4.4%)
Treatment-related questions	63 (34.8%)
Understanding disease progression	42 (23.2%)
Diagnosis seeking	20 (11.0%)
Do I need to go to the ER?	1 (0.6%)
Do you have advice for alleviating this symptom?	8 (4.4%)
Exercise questions	5 (2.8%)
Other	4 (2.2%)

**Table 5:** Breakdown of the 181 posts seeking health-related information by information seeker

	Patient, N (%)	Loved one, N (%)	Total, N
Is this symptom normal?	6 (24.0%)	19 (76.0%)	25
Where do I get help? How do I speak to my doctor?	1 (20.0%)	4 (80.0%)	5
What do these test results mean?	1 (12.5%)	7 (87.5%)	8
Treatment-related questions	29 (46.0%)	34 (54.0%)	63
Understanding disease progression	7 (16.7%)	35 (83.3%)	42
Diagnosis seeking	9 (45.0%)	11 (55.0%)	20
Do I need to go to the ER?	1 (100.0%)	0 (0.0%)	1
Do you have advice for alleviating this symptom?	4 (50.0%)	4 (50.0%)	8
Exercise questions	5 (100.0%)	0 (0.0%)	5
Other	4 (100.0%)	0 (0.0%)	4

**Table 6:** The 63 treatment-related questions were broken down into four categories based on surgical vs. non-surgical questions

Category	N (%)
Pre-op surgical questions	20 (31.7%)
Post-op surgical questions	23 (36.5%)
Non-surgical treatment questions	17 (27.0%)
Non-surgical treatment symptom questions	3 (4.8%)

**Table 7:** Breakdown of the 63 treatment-related questions by Redditors seeking information: patients and loved ones

Category	Patient, N (%)	Loved One, N (%)	Total, N
Pre-op surgical questions	11 (55.0%)	9 (45.0%)	20
Post-op surgical questions	11 (47.8%)	12 (52.2%)	23
Non-surgical treatment questions	6 (35.3%)	11 (64.7%)	17
Non-surgical treatment symptom questions	1 (33.3%)	2 (66.7%)	3

**Table 8:** Sixty-eight social advice posts were further stratified into five different categories

Category	N (%)
Daily activities	31 (45.6%)
Communicating with others	7 (10.3%)
Learning/school	5 (7.4%)
Anxiety/depression	17 (25.0%)
Other	8 (11.8%)

**Table 9:** Sixty-eight social advice posts broken down by author: patient or loved one

Category	Patient, N (%)	Loved one, N (%)	Total, N
Daily activities	11 (35.5%)	20 (64.5%)	31
Communicating with others	0 (0.0%)	7 (100.0%)	7
Learning/school	1 (20.0%)	4 (80.0%)	5
Anxiety/depression	11 (64.7%)	6 (35.3%)	17
Other	1 (12.5%)	7 (87.5%)	8

There were notable trends among the posts themselves. Of the six pregnant moms whose fetuses were diagnosed with spina bifida, three mothers inquired about disease progression, while the other three asked a variety of other health and social-related questions. Within the health advice category, r/scoliosis patients and r/epilepsy loved ones sought the most advice. Notably, within the health advice category, hydrocephalus patients comprised 8 of the 11 (72.7%) postoperative surgical treatment questions, and hydrocephalus loved ones comprised 7 of the 12 (58.3%) of the post-operative surgical treatment questions and, overall, 15 of the 23 (65.2%) post-op surgical treatment questions. Within the social advice category, r/cerebralpalsy comprised the majority (n=12, 38.7%) of all daily activities’ questions (Appendix B, Table 13). Posts ranged widely among activities required to function in society, such as holding down a job, dealing with social scrutiny for appearing differently, and participating in hobbies.

## Discussion

This study provides a published analysis of pediatric neurosurgery patient/loved one activity on a major social media platform, providing neurosurgeons with valuable insight into the questions and concerns our patients face between clinic visits.

Reddit users are seeking and sharing medical information socially [15]. Many are already actively using these platforms to seek out answers to questions on pediatric conditions with potential neurosurgical implications. Adults between the ages of 40-59 are most likely to seek health information online, in part explaining the greater number of loved ones inquiring on Reddit. This can be partially explained by the increasing caretaking role as people age [16]. People report that information found online can help them feel more comfortable about seeking and accepting health advice [16]. The sharing of information from providers on social media platforms like Reddit provides an opportunity to disseminate accurate information to patients and loved ones seeking additional education.

Based on the posts surveyed, 13-17-year-olds are most concerned about disease progression, with the greatest number of postings in this age group belonging to r/hydrocephalus. Hydrocephalus loved ones comprised 7 of the 12 post-operative surgical treatment questions (Appendix B, Table 12). This supports the findings of Elkarim et al., suggesting hydrocephalus patients are actively engaging in online communities to prepare for upcoming hospital visits [7]. With younger patients looking to online forums for answers, healthcare providers need to ensure all questions are being addressed during visits, even when these patients are accompanied by a guardian.

A strong social support network has been shown to reduce stress and help with adjustment in children [17,18]. Among social advice, both patients and loved ones are concerned with daily living activities, and patients are equally concerned with their anxiety and depression. With a quarter of individuals inquiring about mental health, healthcare providers should be equipped with the appropriate information on available/accessible mental health services and community resources (Appendix B, Table 13). Table 3 shows that patients are also turning to Reddit to understand the limits of their physical abilities. Commenters will often suggest exercises they use themselves, however, receiving information from anyone on the internet can potentially do more harm than good, as patients can easily receive advice that is not tailored to their bodies and needs. Healthcare providers need to address these physical concerns and ensure patients are getting referred to the appropriate physical trainers.

With over 34,000 members in the r/epilepsy and r/seizures subreddits, these communities are actively expressing their healthcare opinions on various social media platforms [19]. Our study demonstrates that r/epilepsy loved ones and r/scoliosis patients sought out the most health advice (Appendix B, Table 10). With r/epilepsy loved ones mainly seeking to understand disease progression, healthcare providers have an opportunity to discuss the long-term outlooks of epilepsy. Further, with r/scoliosis patients wanting to understand treatment options, healthcare providers can be more detailed about treatment plans, especially when patients have surgical questions (Appendix B, Table 11).

Individuals with cerebral palsy frequently engage in online communities. Our findings that 45.6% of all activities of daily living questions stemmed from r/cerebralpalsy, support other studies that found how individuals with cerebral palsy enjoy using social media to bypass the constraints of a face-to-face interaction [20]. Understanding how people with disabilities use social media can also help technology developers who create adaptive software, such as augmentative and alternative communication, or communication boards and speech-generating devices, to be better suited to the uses of social media platforms [20]. As social media becomes more and more integrated into our daily lives, providers need to understand how to these platforms to appropriately assess the needs of their patients and to better improve patient-provider communication.

Interestingly, the authors of this study did not identify any obvious misinformation, either in the comments or the posts. Redditors generally understood their knowledge limitations and advised other Redditors to seek further clarification from healthcare providers. Commenters often answered questions from their own perspective and noted that their experience may not relate exactly to the experience of others. Importantly, however, patients and loved ones can potentially be influenced by others to obtain or avoid treatment. Posters occasionally complain that their doctors are not doing everything they can, and a fellow Redditor would encourage the poster to seek a second opinion from a specialist in that field before losing hope. Whether or not there is truth to the poster’s opinion, it’s important to note the power fellow Redditors have in encouraging others to take charge of their own health care. Perhaps that is one glaring reason people are turning to Reddit to ask questions to ensure they are doing everything they can to help themselves. Additionally, patients are seeking more information about their treatments (n=29, Table 4) by asking questions about adverse effects and if symptoms are normal (n=6, Table 5). Commenters sharing similar symptoms can motivate posters to adhere to their treatment and promote medication adherence through a sense of comradery while also encouraging posters to seek health advice when they believe the symptom is out of the ordinary. The influence a community of shared experiences has on patient care should not be underestimated. Healthcare providers can use this information to better educate patients on when it’s important to seek a second opinion or to see a doctor for a symptom they are experiencing, whether it’s from an adverse effect or a worrisome disease symptom.

These findings can help neurosurgical providers prepare for patient/family interviews in a clinical setting. Future studies could further explore the different communities on Reddit on other popular social media websites with community forums, such as Facebook, Twitter, and YouTube, and dive deeper into topics in specific communities, such as common post-operative hydrocephalus questions. Demographics may help further segregate the tailoring of information dissemination. Health professionals "controlling the narrative" in these communities may also lead to the dissemination of life-saving information, such as warning signs of diseases and treatments.

Limitations

The anonymity of Reddit makes it difficult to verify the information shared among users. It is not possible to confirm the number of active users out of the total followers in each subreddit due to the anonymity of Reddit. Because some original posters (OP) did not specify their age, more posts could have potentially been included, especially where Redditors indicated that they were a "teenager." Further, we cannot confirm how many members of the subreddits were children with the medical condition or their loved ones, therefore the total number of Redditors in these subreddits does not necessarily represent our target study population. Additionally, the anonymity of Reddit prevented the identification of the demographics of patients and loved ones. Being able to further determine which demographics of patients were using Reddit as a resource would create a more thorough analysis of the kinds of patients and loved ones who are accessing these resources. It may be the case that certain groups have less access, thus focusing efforts on information distribution on this resource would prove ineffective in adequately addressing all aspects of the patient population, leading to poorer health outcomes for such groups.

The OPs themselves may not necessarily represent the subreddit's user population and may skew the data to reflect some common and unknown characteristics of the Redditors who post to the subreddit as opposed to those who do not. While picking the posts from the "hot" tab allows for those posts that are the most popular in the group, these posts still may not adequately represent the experiences or questions of the entire group.

## Conclusions

Reddit is a social media avenue clinicians could use to track and disseminate correct information to current and prospective subpopulations of pediatric patients and their loved ones. Healthcare providers can use the information gathered to understand some themes and better tailor their discussions with parents and pediatric patients. Providers should ensure that both pediatric patients and their parents have their concerns addressed and explain physical limitations prior to leaving the office so that patients don’t have to turn to the internet. Future research could dive into these specific disease communities and understand other commonly asked questions among other popular social media platforms, such as X, formerly known as Twitter and Facebook.

## Appendices

Appendix A

The following search terms were used to generate a list of Subreddits: aneurysm, arachnoid cyst, arteriovenous malformation, ataxia, arteriovenous malformation, AVM, brain cancer, brain tumor, cavernous angioma, cavernous malformation, cerebral palsy, chiari malformation, deep brain stimulation, dermoid cyst, epilepsy, headaches, herniated disc, hydrocephalus, kyphosis, lordosis, migraines, moyamoya, muscular dystrophy, neurosurgery, pineal cyst, sacral dimples, scoliosis, seizures, spina bifida, spinal cord, stroke, syringomyelia, syrinx, traumatic brain injury, ventriculoperitoneal shunt, VP shunt.

Appendix B

**Table 10:** Categorization of the 286 posts by subreddit

Subreddit	Asking for social advice		Asking for health advice		Providing health education/suggesting equipment		Personal experience/progress post		Other	
	Patient, N	Loved one, N	Patient, N	Loved one, N	Patient, N	Loved one, N	Patient, N	Loved one, N	Patient, N	Loved one, N
r/epilepsy	4	6	7	28	0	1	1	3	0	0
r/scoliosis	7	3	29	7	0	0	3	1	0	0
r/cerebralpalsy	7	15	2	19	0	0	0	3	1	3
r/seizures	0	3	4	13	0	0	2	2	0	0
r/braintumor	0	1	4	3	0	1	2	3	0	0
r/hydrocephalus	1	7	14	24	0	1	2	1	0	0
r/spina bifida	5	9	3	18	0	1	1	2	0	0
r/degendiscdisease	0	0	2	0	0	0	0	0	0	0
r/chiarimalformation	0	0	0	1	0	0	1	1	0	0
r/brainaneurysm	0	0	2	0	0	0	0	0	0	0
r/ataxia	0	0	0	1	0	0	0	0	0	0
r/herniateddisc	0	0	0	0	1	0	0	0	0	0
TOTAL	24	44	67	114	1	4	12	16	1	3
OVERALL TOTAL	68		181		5		28		4	

**Table 11:** Breakdown of the 181 health advice questions asked by either a patient or a loved one from the included subreddits Pt: Patient; L: Loved one

Subreddit	Is this a normal symptom?		Where can I get help?		What do these test results mean?		Disease progression questions		Treatment questions		Diagnosis seeking		Do I need to see a doctor?		How can I alleviate my symptoms?		Exercise-related questions		Other	
	Pt, N	L, N	Pt, N	L, N	Pt, N	L, N	Pt, N	L, N	Pt, N	L, N	Pt, N	L, N	Pt, N	L, N	Pt, N	L, N	Pt, N	L, N	Pt, N	L, N
r/epilepsy	0	3	0	3	0	0	2	11	0	7	3	3	0	0	0	1	0	0	2	0
r/scoliosis	4	0	0	0	0	0	3	3	13	3	0	0	0	0	4	1	4	0	1	0
r/cerebralpalsy	0	4	0	1	0	1	0	3	2	6	0	4	0	0	0	0	0	0	0	0
r/seizures	0	4	0	0	0	3	0	2	1	1	2	3	1	0	0	0	0	0	0	0
r/braintumor	0	0	0	0	0	1	1	2	0	0	2	0	0	0	0	0	0	0	1	0
r/hydrocephalus	2	5	0	0	1	2	1	8	9	9	0	0	0	0	0	0	1	0	0	0
r/spina bifida	0	3	1	0	0	0	0	5	2	8	0	0	0	0	0	2	0	0	0	0
r/degendiscdisease	0	0	0	0	0	0	0	0	2	0	0	0	0	0	0	0	0	0	0	0
r/chiarimalformation	0	0	0	0	0	0	0	1	0	0	0	0	0	0	0	0	0	0	0	0
r/brainaneurysm	0	0	0	0	0	0	0	0	0	0	2	0	0	0	0	0	0	0	0	0
r/ataxia	0	0	0	0	0	0	0	0	0	0	0	1	0	0	0	0	0	0	0	0
r/herniateddisc	0	0	0	0	0	0	0	0	0	0	0	0	0	0	0	0	0	0	0	0
TOTAL	6	19	1	4	1	7	7	35	29	34	9	11	1	0	4	4	5	0	4	0
OVERALL TOTAL	25		5		8		42		63		20		1		8		5		4	

**Table 12:** Categorization of the 63 treatment-related questions asked by patients or their loved ones

Subreddit	Surgical treatment questions		Post-surgical treatment symptom questions		Non-surgical treatment questions		Non-surgical treatment symptom questions	
	Patient, N	Loved one, N	Patient, N	Loved one, N	Patient, N	Loved one, N	Patient, N	Loved one, N
r/epilepsy	0	0	0	0	0	5	0	2
r/scoliosis	7	1	3	2	3	0	0	0
r/cerebralpalsy	0	2	0	0	2	4	0	0
r/seizures	0	0	0	0	1	1	0	0
r/braintumor	0	0	0	0	0	0	0	0
r/hydrocephalus	1	2	8	7	0	0	0	0
r/spina bifida	2	4	0	3	0	1	0	0
r/degendiscdisease	1	0	0	0	0	0	1	0
r/chiarimalformation	0	0	0	0	0	0	0	0
r/brainaneurysm	0	0	0	0	0	0	0	0
r/ataxia	0	0	0	0	0	0	0	0
r/herniateddisc	0	0	0	0	0	0	0	0
TOTAL	11	9	11	12	6	11	1	2
OVERALL TOTAL	20		23		17		3	

**Table 13:** Categorization of the 68 social advice posts asked by patients or their loved ones

Subreddit	Daily activities		How to speak to other people/child about condition		Learning/school		Anxiety/depression		Other	
	Patient, N	Loved one, N	Patient, N	Loved one, N	Patient, N	Loved one, N	Patient, N	Loved one, N	Patient, N	Loved one, N
r/epilepsy	0	3	0	1	1	0	3	2	0	0
r/scoliosis	2	2	0	0	0	0	4	0	1	1
r/cerebralpalsy	5	7	0	2	0	1	2	1	0	4
r/seizures	0	2	0	0	0	0	0	1	0	0
r/braintumor	0	0	0	0	0	1	0	0	0	0
r/hydrocephalus	1	4	0	0	0	2	0	1	0	0
r/spina bifida	3	2	0	4	0	0	2	1	0	2
r/degendiscdisease	0	0	0	0	0	0	0	0	0	0
r/chiarimalformation	0	0	0	0	0	0	0	0	0	0
r/brainaneurysm	0	0	0	0	0	0	0	0	0	0
r/ataxia	0	0	0	0	0	0	0	0	0	0
r/herniateddisc	0	0	0	0	0	0	0	0	0	0
TOTAL	11	20	0	7	1	4	11	6	1	7
OVERALL TOTAL	31		7		5		17		8	
